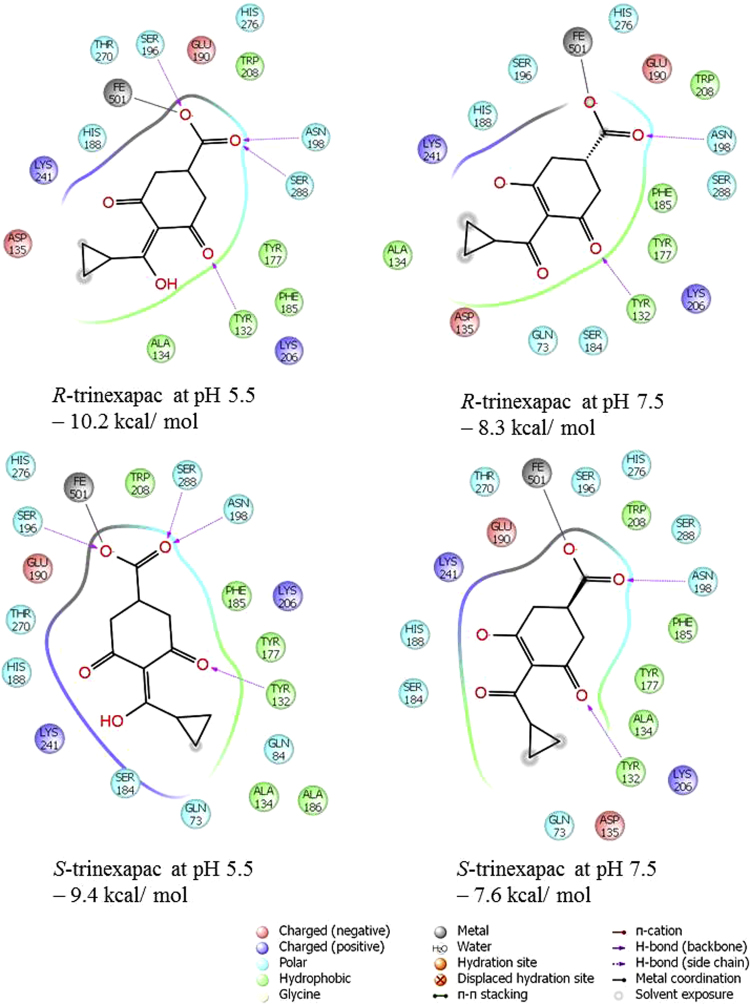# Prohexadione, a plant growth regulator, inhibits histone lysine demethylases and modulates epigenetics

**DOI:** 10.1016/j.toxrep.2014.10.026

**Published:** 2014-11-04

**Authors:** Divya Teja Vavilala, Sujatha Reddy, Sachchidanand, Swami Prakash, V.K. Chaithanya Ponnaluri, Arvind Kumar, Mridul Mukherji

**Affiliations:** a Division of Pharmaceutical Sciences, School of Pharmacy, University of Missouri–Kansas City, Kansas City, MO 64108, USA; b Centre for Cellular and Molecular Biology, Hyderabad 500007, Andhra Pradesh, India; c National Institute of Pharmaceutical Education & Research (NIPER), Hajipur 844101, Bihar, India

**Keywords:** Epigenetics, Histone lysine demethylase, Jmjd2a, Herbicide, Prohexadione, Neural stem cells

## Abstract

**Background:**

Epigenetic modifications, particularly DNA methylation and posttranslational histone modifications regulate heritable changes in transcription without changes in the DNA sequence. Despite a number of studies showing clear links between environmental factors and DNA methylation, little is known about the effect of environmental factors on the recently identified histone lysine methylation. Since their identification numerous studies have establish critical role played by these enzymes in mammalian development.

**Objectives:**

Identification of the Jumonji (Jmj) domain containing histone lysine demethylase have added a new dimension to epigenetic control of gene expression by dynamic regulation of histone methylation marks. The objective of our study was to evaluate the effect of prohexadione and trinexapac, widely used plant growth regulators of the acylcyclohexanediones class, on the enzymatic activity of histone lysine demethylases and histone modifications during the neural stem/progenitor cell differentiation.

**Methods:**

Here we show that prohexadione, but not trinexapac, directly inhibits non-heme iron (II), 2-oxoglutarate-dependent histone lysine demethylase such as Jmjd2a. We used molecular modeling to show binding of prohexadione to Jmjd2a. We also performed *in vitro* demethylation assays to show the inhibitory effect of prohexadione on Jmjd2a. Further we tested this molecule in cell culture model of mouse hippocampal neural stem/progenitor cells to demonstrate its effect toward neuronal proliferation and differentiation.

**Results:**

Molecular modeling studies suggest that prohexadione binds to the 2-oxoglutarate binding site of Jmjd2a demethylase. Treatment of primary neural stem/progenitor cells with prohexadione showed a concentration dependent reduction in their proliferation. Further, the prohexadione treated neurospheres were induced toward neurogenic lineage upon differentiation.

**Conclusions:**

Our results describe an important chemico-biological interaction of prohexadione, in light of critical roles played by histone lysine demethylases in human health and diseases.

## 1 Introduction

Epigenetics dictates the foundation of cellular growth, differentiation, and identity. They are regulated by covalent modifications of the genomic DNA, particularly methylation at carbon-5 of cytosine residues located in the CpG islands, and post-translational modifications of histones. A number of exogenous factors can influence the cellular epigenetics and cause heritable changes in gene expression without changing the genomic DNA sequence by manipulating the cellular DNA methylation patterns. Results from a number of studies have established an association between DNA methylation and environmental metals including cadmium, lead, nickel, and arsenic [1], [2]. In addition, environmental chemicals such as trichloroethylene, dichloroacetic acid, trichloroacetic acid, benzene, *etc.* can also influence epigenetics by changing the DNA methylation [3], [4], [5].

Eukaryotic histones, around which the genomic DNA is wrapped, also undergo extensive post-translational modifications which regulate epigenetics by controlling the accessibility and usage of the genomic DNA. As a result, histone modifying enzymes, specifically those that modulate acetylation and methylation, play a vital role in the transcriptional regulation of genes. Histones are methylated on the lysine or arginine residues. The predominant sites of lysine methylation include histone-3 lysine-4 (H3-K4), H3-K9, H3-K27, H3-K36, H3-K79 and H4-K20 [6]. For a long time, histone methylation marks were considered to be static. However, identification of lysine-specific demethylase 1 (LSD1, which can only demethylate mono- and di-methylated H3-K4 and H3-K9) and a number of Jumonji (Jmj) domain containing iron (II), 2-oxoglutarate (2OG)-dependent histone lysine demethylases (KDMs, which can even demethylate tri-methylated lysine residues of histone) have added a new dimension to the dynamic epigenetic regulation [7]. Despite a number of studies showing clear links between environmental factors and DNA methylation, little is known about the effect of environmental factors on histone lysine methylation.

Prohexadione (3,5-dioxo-4-propionylcyclohexanecarboxylic acid) and trinexapac [4-(cyclopropylhydroxymethylene)-3,5-dioxocyclohexanecarboxylic acid] are plant growth regulators (PGRs) of the acylcyclohexanediones class. Trinexapac–ethyl (an ester form, also known as Primo/Cimectacarb/Cimetacarb) is one of the most commonly used PGR on fine turf surfaces throughout the world; while prohexadione–calcium (a salt form, also known as Apogee/Baseline) inhibits the synthesis of gibberellins, a naturally occurring plant hormone, and is a widely used chemical for controlling vegetative growth. It is also sprayed on apple and pear leaves, which inhibits flavanone 3β-hydroxylase and flavonol synthase resulting in changes in the flavonoid spectrum. This results in an enhanced resistance of apple and pear leaves toward two major pome fruit diseases, fire blight (caused by *Erwinia amylovora*) and apple scab (caused by *Venturia inaequalis*) [8], [9]. Due to structural similarities between prohexadione and trinexapac to 2OG, it has been proposed that acylcyclohexanediones such as prohexadione enhance resistance by inhibiting iron (II), 2OG-dependent dioxygenases (*e.g.* flavanone 3β-hydroxylase and flavonol synthase) which play important roles in flavonoid biosynthesis [10]. Therefore, we hypothesized that these two PGRs may inhibit iron (II), 2OG-dependent KDMs and modulate epigenetics in mammalian cells. Here, we provide evidence that prohexadione, but not trinexapac, potently inhibits KDMs and modulates epigenetics in cell-based studies.

## 2 Materials and methods

### 2.1 Preparation of Jmjd2a protein and ligands for docking studies

The Jmjd2a protein has been crystallized at pH 5.5, and the structure was solved at 2.15 Å resolution [11]. This X-ray crystal structure of Jmjd2a protein (PDB code: 2OQ7) was used for docking studies. This structure of Jmjd2a protein represents a catalytically inactive enzyme since the normal cofactors iron (II) and 2OG were replaced by Ni(II) and *N*-oxalylglycine, a competitive inhibitor of 2OG-dependent dioxygenases. Therefore, the inhibitory Ni(II) was replaced by iron (II) in the active site, and the Jmjd2a protein preparation for docking studies was carried out using protein preparation wizard (PPW) of Schrodinger's Suite 2012. The water molecules were removed from this structure, and the “het states” for the iron (II) and *N*-oxalylglycine were generated at pH 5.5 (pH at which crystallization was carried out) and pH 7.5 (pH at which Jmjd2a enzymatic assays were carried out in this study) using Epik [12], [13]. Epik is a program which predicts the p*K*_a_ values of ionizable groups in small molecules/ligands (*e.g. N*-oxalylglycine, prohexadione *etc.*) at a pH or within a pH range. In the refinement stage of PPW, all the added hydrogen atoms in the prepared structure of the Jmjd2a protein were minimized, and the H-bond optimization was carried out using protonation states of residues at pH 5.5 and 7.5. The p*K*_a_ values of amino acid residues at a given pH were calculated using PROPKA [14]. Finally, a restrained minimization of Jmjd2a structure was carried out using OPLS 2005 force field.

For the preparation of ligands for docking studies, the two-dimensional (2D) structures of *N*-oxalylglycine, prohexadione, and trinexapac were drawn. These 2D structures were converted to 3D structures, which generated *R/S*-stereoisomers of prohexadione and trinexapac, at pH 5.5 and 7.5 using ligprep (LigPrep, version 2.5, Schrödinger, LLC, New York, NY, 2012) and Epik (Epik, version 2.3, Schrödinger, LLC, New York, NY, 2012).

### 2.2 Docking of ligands to Jmjd2a protein

Ligands prepared at pH 5.5 and 7.5 were docked to Jmjd2a protein prepared at pH 5.5 and 7.5, respectively. A docking region, also known as the grid, was centered on the template ligand (*i.e. N*-oxalylglycine of the prepared Jmjd2a protein) with a default box size of 26 Å × 21 Å × 24 Å. The docking calculations were carried out using Glide in the extra precision (XP) mode [15]. Glide does flexible ligand docking into a rigid protein structure by sampling of positional, orientational, and conformational degrees of freedom of the ligand.

### 2.3 Cloning, expression and purification of Jmjd2a

Jumonji domain containing region of *Jmjd2a* gene was cloned, expressed, and purified as described earlier [16], [17], with minor modifications. In brief, the N-terminal GST tag containing fusion Jmjd2a enzyme in pGEX-4T1 expression vector (GE Healthcare, Piscataway, NJ) was purified from *Escherichia coli* BL21 (DE3) cells, using affinity chromatography. The chromatographic fractions containing purified Jmjd2a enzyme was dialyzed in 25 mM NaCl (Sigma–Aldrich, St. Louis, MO), 25 mM HEPES (Sigma–Aldrich), pH 7.5 for ≈8 h. The dialyzed Jmjd2a protein was stored in 15% glycerol at –80 °C.

### 2.4 *In vitro* demethylation assay

The *in vitro* Jmjd2a demethylation assays were carried out in triplicates as described earlier [11]. All the assays were carried out in 50 μl reaction volume. The *in vitro* reactions were performed in 25 mM HEPES buffer at pH 7.5 by adding the substrate solution to the enzyme solution and incubating for 30 min. The enzyme solution contained 2 μM of purified Jmjd2a, 3 μM FeSO_4_ and 20 μM ascorbate in 25 mM HEPES buffer and the substrate solution contained 6 μM 2OG and 10 μM of the peptide substrate in 25 mM HEPES buffer. The enzyme solution was incubated at room temperature for 15 min in the absence or presence of 1 mM inhibitors *i.e. N*-oxalylglycine (Frontier Scientific, Logan, UT), prohexadione (Chem Service, West Chester, PA) and trinexapac (Crescent Chemical Company, Islandia, NY) before the substrate solution was added. The reaction was stopped by adding 50 μl of methanol, followed by the addition of 100 μl of 80 mM tri-ammonium citrate. Further, the reaction mixture was centrifuged using an Eppendorf 5417C centrifuge at 13,000 rpm for 2 min.

### 2.5 Demethylation assay analysis using matrix associated laser desorption ionization-time of flight (MALDI-TOF)

**Fig. 1:**
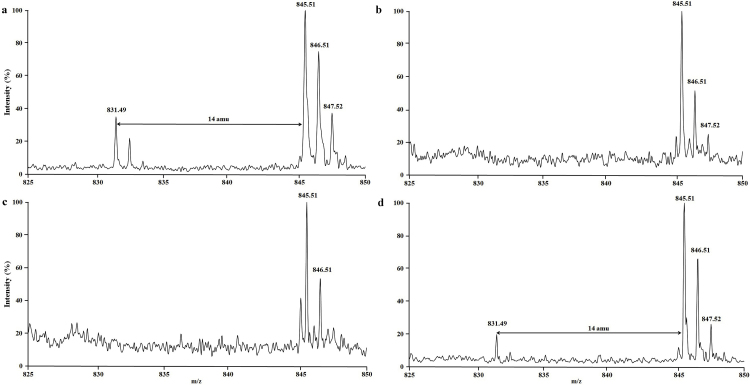
Mass spectra (MALDI-TOF) of Jmjd2a catalyzed demethylation reactions using H3-K9me3 peptide as substrate in the absence (a), or presence of *N*-oxalylglycine (b), prohexadione (c), and trinexapac (d).

The supernatant (5 μl) from the above reaction mixture was added to 5 μl of the matrix *i.e.* α-cyano-4-hydroxycinnamic acid (CHCA, Sigma–Aldrich). From the above mixture, 1 μl was spotted in triplicates on a MALDI plate (pre-spotted with 1 μl of matrix) for analysis using a MALDI-TOF instrument. All spectra were collected on a Voyager DE PRO MALDI-TOF mass spectrometer (Applied Biosystems, Foster City, CA). Spectra for each sample was obtained by averaging 500 laser shots. Data were collected in triplicates to capture the variability related to demethylation reaction, sample preparation, data collection, and data extraction during MALDI analysis. Only one representative spectrum under each assay condition (*e.g.* with or without inhibitor) is shown in Fig. 1.

### 2.6 Mouse neural stem/progenitor cell culture, neurosphere imaging and analysis

Mouse hippocampal neural stem/progenitor cells (NSCs/NPCs) were harvested and cultured according to our previous study [18]. Briefly, postnatal day 3 (P3) C57BL/6 female mice pups were euthanized by decapitation and hippocampi were dissected out, minced, and triturated in 0.025% trypsin–EDTA for 7 min at 37 °C. Activity of trypsin was arrested by the addition of 0.014% trypsin inhibitor containing 1 mg/ml DNase-1 (Gibco, Carlsbad, CA). The isolated cells were pelleted down at 700 × *g* for 5 min and resuspended in NeuroCult NSC basal media (Stemcell Technologies, Vancouver, BC, Canada). The basal media contained 1× mouse proliferative supplement (Stemcell Technologies), 100 U/ml penicillin, 40 μg/ml streptomycin, 0.02% BSA (Calbiochem, San Diego, CA), 10 ng/ml basic fibroblast growth factor (Sigma–Aldrich), 20 ng/ml epidermal growth factor (Calbiochem), and 0.04 mg/ml heparin (Sigma–Aldrich). Single cell suspensions were obtained by passing the resuspended cells through a 40 μm filter. Isolated cells were seeded in a 96-well plate to form neurospheres. Prohexadione and trinexapac–ethyl (Chem Service, West Chester, PA) were dissolved in DMSO and sterile water, respectively. For the cell-based studies trinexapac–ethyl, instead of trinexapac, was used to enhance its cell permeability. After its transport inside the cells, it is de-esterified by cellular esterases [19], to generate trinexapac. Neurosphere cultures were treated with 1, 1.5, and 2 mM of PGRs along with solvent controls for a period of 6 days. At the end of day 6, neurospheres were imaged using Zeiss Axiovert 200 M Live Cell Workstation. The size and the number of neurospheres were analyzed using Axiovision LE Rel 4.3 software by taking images of four random fields per well at 10× magnification. The neurospheres were plated in chambered cover glass (ThermoFisher Lab-Tek, Waltham, MA) and induced for differentiation into neurons and glia by transferring them to differentiation medium. The differentiation media contsisted of Neurocult NSC basal medium with 1% FBS (Gibco), 100 U/ml penicillin, 40 μg/ml streptomycin, and 0.02% BSA. PGRs along with solvent controls were added in the differentiation media. The differentiation was induced for 5 days, after which images of four random fields per well at 10× magnification were captured. The sizes and numbers of neurospheres were analyzed using Axiovision LE Rel 4.3 software. All animal procedures were approved by the Institutional Animal Ethics Committee (IAEC) of the Centre for Cellular and Molecular Biology (CCMB), Hyderabad, Andhra Pradesh, India (IAEC/CCMB/Protocol No. 25/2011).

### 2.7 Immunofluorescence studies

Neurospheres upon differentiation were immunostained using standard procedures. Briefly, cells were fixed with 4% paraformaldehyde in 1× PBS for 10 min and permeabilized with 1× PBS containing 0.3% Triton X-100 for 90 min. After treating the differentiated cells in the blocking solution (5% BSA in 1× PBS containing 0.3% TritonX-100) for 2 h at room temperature, cells were incubated overnight at 4 °C in the blocking solution containing following primary antibodies: mouse anti-neuronal nuclei or NeuN (Millipore, Billerica, MA) at 1:100 dilution, rabbit anti-glial fibrillary acidic protein or GFAP (Abcam, Cambridge, MA) at 1:1000 dilution, rabbit anti-H3-K9me2 (Millipore) at 1:1000 dilution, rabbit anti-H3-K27me2 (Abcam) at 1:1000 dilution, and rabbit anti-H3-K36me2 (Abcam) at 1:1000 dilution. After 3 washes with 1× PBS, cells were incubated with appropriate secondary antibodies: Cy3 conjugated goat anti-rabbit IgG (Abcam) at 1:1000 dilution and Alexafluor 488 conjugated goat anti-mouse IgG (Life Technologies, Grand Island, NY) at 1:500 dilution.

### 2.8 Statistical analysis

Students's *t*-test was performed to evaluate the strength of significance. To evaluate the effect of prohexadione treatment on neural stem/progenitor cells (NSCs/NPCs) proliferation and/or differentiation, the ‘Fisher's Exact’ statistical test was performed because the sample size (number of experimental replicates) was less than ten. This analysis was performed to evaluate the neurosphere size distribution in each experimental group. The total number of neurospheres were considered as 100%. *P* values less than 0.05 were considered as significant difference. All statistical analysis was carried out using GraphPad Prism Software.

## 3 Results and discussions

### 3.1 Prohexadione and trinexapac bind at the 2OG binding site of Jmjd2a demethylase

Due to structural similarities between 2OG, prohexadione, and trinexapac it has been proposed that prohexadione and trinexapac act as competitive inhibitors of 2OG-dependent enzymes in the gibberellin biosynthetic pathway. Therefore, we hypothesized that prohexadione and trinexapac may bind at the active site of recently characterized KDMs. In humans ∼25–30 putative Jmj domain containing iron (II), 2OG-dependent KDMs have been identified that are classified into 7 families based on their sequences [6], [7]. Since the protein purification, enzymatic assay, and crystal structure of the jumonji domain-2 (Jmjd2) family KDMs are documented in the literature [11], [16], [17], we focused on Jmjd2a isoform as a representative KDM for docking and *in vitro* enzymatic studies.

For *in silico* experiments, the 3D output structures of ligands (*e.g. N*-oxalylglycine, prohexadione, and trinexapac) generated at pH 5.5 and 7.5 (Fig. S1), were docked to the Jmjd2a protein prepared at pH 5.5 and 7.5, respectively. The output structures of *N*-oxalylglycine at both pH 5.5 and 7.5 were the same. Docking of the ligands at the Jmjd2a active site gave the best docking scores (–11.5 kcal/mol and −9.6 kcal/mol at pH 5.5 and 7.5, respectively) for *N*-oxalylglycine, which is structurally similar to Jmjd2a co-substrate/natural ligand, 2OG. Since the crystal structure of the substrate bound Jmjd2a demethylase was solved with 2OG structural analog, *N*-oxalylglycine (instead of 2OG [11], to trap the enzyme in an inactive form), for comparison we performed our docking experiments with *N*-oxalylglycine and not 2OG. The docking pose of *N*-oxalylglycine was very similar to its co-crystallized structure with Jmjd2a [11] (Fig. S2), validating our docking protocol.

A conversion of 2D input structures of prohexadione and trinexapac into 3D output structure generated *R/S*-stereoisomers (Fig. S1). It is important to note that both prohexadione and trinexapac are available and used in the environment as racemic mixtures containing both *R/S*-stereoisomers. Therefore, we performed our docking experiments with both the enantiomers. Although, *N*-oxalylglycine binds to iron (II) at the Jmjd2a catalytic site in a bidentate fashion, both the stereoisomers of prohexadione and trinexapac bind in a monodentate manner at pH 5.5 and 7.5 (Figs. S3–S5). This may account for lower docking scores for prohexadione and trinexapac stereoisomers, compared to *N*-oxalylglycine, at both pH 5.5 and 7.5 (Table 1). The docking scores of all the ligands were lower at pH 7.5 compared to pH 5.5. This could be due to the electrostatic award which was more favorable when the ligands were docked to the Jmjd2a protein prepared at pH 5.5. In general, docking scores and ligand efficiency (*i.e.* docking score/number of heavy atoms) were best for N-oxalylglycine followed by prohexadione (*R*-prohexadione –10.1 kcal/mol and –8.2 kcal/mol at pH 5.5 and 7.5, respectively; *S*-prohexadione –10.3 kcal/mol and –9.3 kcal/mol at pH 5.5 and 7.5, respectively) and then by trinexapac (*R*-trinexapac –10.2 kcal/mol and –8.3 kcal/mol at pH 5.5 and 7.5, respectively; *S*-trinexapac –9.4 kcal/mol and –7.6 kcal/mol at pH 5.5 and 7.5, respectively) (Table 1). Our docking experiments suggest that prohexadione and trinexapac, to a lesser extent, may inhibit Jmjd2a, and possibly other KDMs, by directly binding at the 2OG binding site in the active site of KDMs.

**Table 1:** Name of ligands used for docking experiments using ligprep and Epik with their docking scores at the indicated pH values.

Ligand name	Docking score at pH 5.5	Docking score at pH 7.5
*N*-oxalylglycine	−11.5 kcal/mol	−9.6 kcal/mol
*R*-prohexadione	−10.1 kcal/mol	−8.2 kcal/mol
*S*-prohexadione	−10.3 kcal/mol	−9.3 kcal/mol
*R*-trinexapac	−10.2 kcal/mol	−8.3 kcal/mol
*S*-trinexapac	−9.4 kcal/mol	−7.6 kcal/mol

### Prohexadione inhibits the activity of Jmjd2a demethylase *in vitro*

Jmjd2a demethylates tri-methylated H3-K9 (H3-K9me3), H3-K9me2, and H3-K36me3 [11]. In order to test our hypothesis that prohexadione and trinexapac act as general inhibitors of recently identified KDMs, Jmjd2a was purified to homogeneity and assayed for the ability of different chemicals *e.g. N*-oxalylglycine, prohexadione, and trinexapac to inhibit the demethylation of H3-K9me3 into the dimethylated product, H3-K9me2. The results showed that in the absence of *N*-oxalylglycine and PGRs, Jmjd2a efficiently converted H3-K9me3 peptide into H3-K9me2 (Fig. 1a). However, in the presence of 1 mM *N*-oxalylglycine, a known inhibitor of iron (II), 2OG-dependent KDMs, no product formation was detected (Fig. 1b). These results suggest that our assay conditions are suitable for inhibition studies using prohexadione and trinexapac. The presence of 1 mM prohexadione in the reaction mixture completely abrogated the conversion of H3-K9me3 peptide substrate into H3-K9me2 product (Fig. 1c). However, under the same assay condition only a partial inhibition of Jmjd2a catalytic activity was observed by trinexapac (Fig. 1d). Although our studies were performed with the racemic mixture of trinexapac, which contains both *R*/*S*-stereoisomers, a limited inhibition by trinexapac could be due to poor the docking score, especially for the *S*-trinexapac (–7.6 kcal/mol) at pH 7.5, at which the enzymatic assays were performed. These results demonstrate that prohexadione, and trinexapac to some extent, directly inhibit the catalytic activity of Jmjd2a demethylase.

### 3.2 Prohexadione inhibits the proliferation of neurospheres

**Fig. 2:**
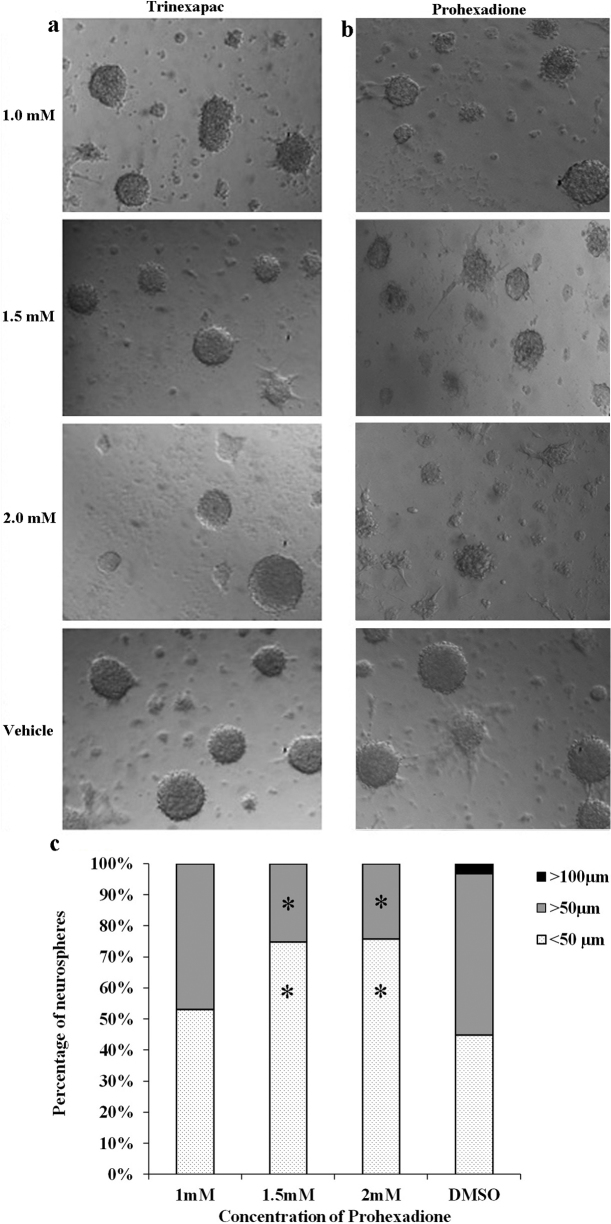
Live-cell imaging of neurospheres treated with different concentrations of prohexadione (a), trinexapac–ethyl (b), and the size-range of neurospheres treated with different concentrations of prohexadione (c). Fisher's exact test was performed to evaluate the effects of prohexadione on the proliferation of neural stem/progenitor cells. *P* values less than 0.05 were considered as significant difference compared to DMSO/vehicle treated control sample and marked as *.

Next, the effects of prohexadione and trinexapac on KDMs were evaluated using neurosphere cultures. Neurospheres are used to study the proliferation, self-renewal, and multi-potency of NSCs/NPCs [20], where KDMs play critical roles [21], [22], [23]. For example, formation of large neurospheres reflects good neurogenic potential of NSCs/NPCs [24]. Therefore, the mouse NSCs/NPCs were treated with different concentrations of prohexadione and trinexapac, and the proliferation of neurospheres were measured. For these studies, the sizes of neurospheres were divided into three different groups: small (<50 μm), medium (50–100 μm), and large (>100 μm). In the DMSO treated control samples 44.93% neurospheres were small, 51.89% were medium, and 3.17% were large in size. Consistent with the results of our docking and *in vitro* enzymatic experiments, trinexapac treated NSCs/NPCs did not show any apparent change in the number, morphology, or size of neurospheres (Fig. 2a). However, with an increase in the prohexadione concentration, the size distribution of neurospheres were 53.14% small and 46.85% medium at 1 mM; 74.83% small and 25.16% medium at 1.5 mM; and 75.81% small and 24.18% medium at 2 mM (Figs. 2b and c). Interestingly, large neurospheres normally seen in neurosphere assays, 3.17% in this case, were completely absent from the prohexadione treated groups, while the numbers of neurospheres in the smaller size range were elevated, indicating an inhibition of neurosphere proliferation (Fig. 2c). Thus, consistent with our docking and biochemical studies, administration of selected PGRs of the acylcyclohexanediones class had different effect on the growth of neural stem/progenitor cells (NSCs/NPCs), as shown in Fig. 2. Trinexapac, which doesn’t block the Jmjd2a demethylase activity, fails to affect the growth potential of NSCs/NPCs. On the other hand prohexadione, which blocks the Jmjd2a demethylase activity, significantly reduces the growth potential of NSCs/NPCs in a dose dependent manner (Fig. 2). Taken together, our results indicate a clear correlation between the inhibition of demethylase activity and the stem cell growth by selected PGRs.

### 3.3 Prohexadione acts by increasing the methylation on H3-K27 and H3-K36 residues

**Fig. 3:**
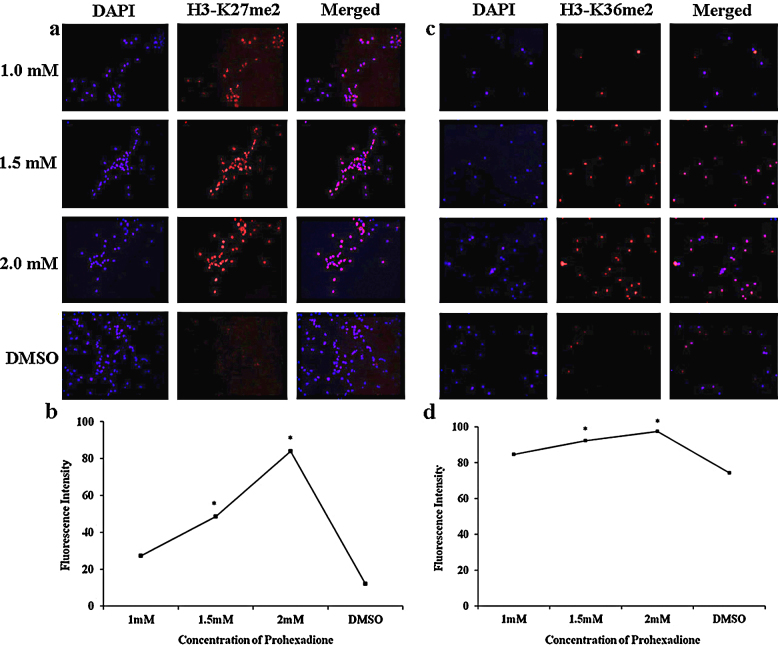
Immunofluorescence staining for H3-K27me2 in differentiated neural stem cell cultures treated with prohexadione (a), immunofluorescence staining for H3-K36me2 in differentiated neural stem cell cultures treated with prohexadione (b). Graphical representation of immunofluorescence staining for H3-K27me2 (c), and H3-K36me2 (d) in differentiated neural stem cell cultures treated with various dose of prohexadione.

**Fig. 4:**
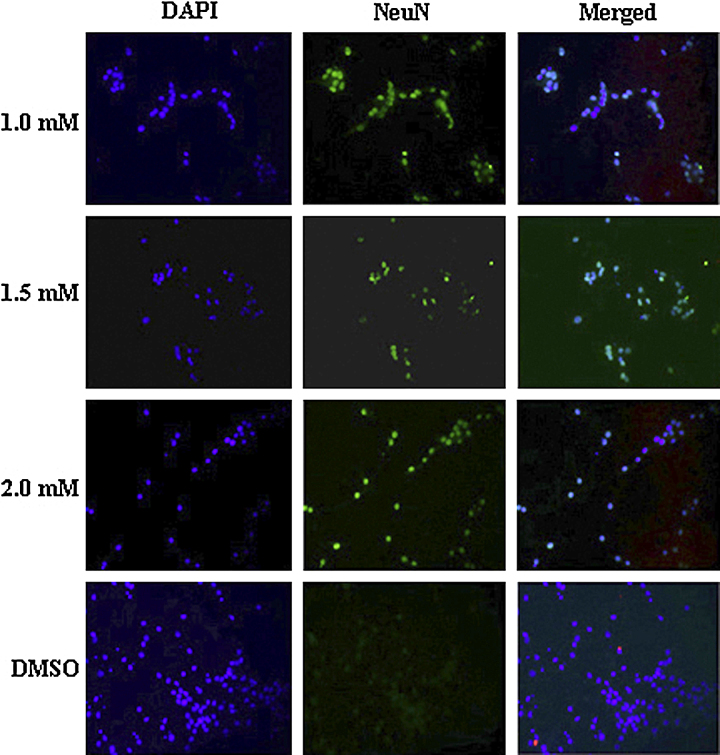
Immunofluorescence staining for the neuronal mark NeuN upon differentiation of the neural stem/progenitor cell cultures treated with prohexadione.

Finally, we evaluated if prohexadione-mediated inhibition of neurosphere proliferation is mediated *via* inhibition of demethylation on H3-K9, H3-K27 and H3-K36 sites by immunofluorescence studies. To this end, no significant change was observed in the methylation status of H3-K9me2 mark (data not shown); however, the H3-K27me2 and H3-K36me2 levels increased with an increase in the concentration of prohexadione (Fig. 3). These studies indicate that prohexadione likely acts *in vivo* by inhibiting H3-K27 and H3-K36 specific demethylases (*e.g.* Jmjd3 and Jmjd2a) [25]. Since the dynamic histone lysine methylations, particularly of H3-K27 residue, play critical roles in neural stem cell proliferation, stem-ness and differentiation [21], [22], [23], we evaluated the cellular fate of prohexadione treated neurospheres by immunofluorescence studies using antibodies for neuronal nuclei or NeuN, a neuronal marker, and for glial fibrillary acidic protein or GFAP, a glial marker. Interestingly, these studies showed that prohexadione treated neurospheres had a concentration dependent increase in the expression of NeuN (Fig. 4), suggesting that these neurospheres were induced toward neurogenic lineage upon differentiation. Taken together, our results establish that prohexadione modulates proliferation and differentiation of neurospheres possibly by acting as a general inhibitor of histone lysine demethylases.

## 4 Conclusion

According to the World Health Organization annually more than 13 million deaths are attributed to environmental causes, and ≈24% of the diseases caused by environmental pollutants can be avoided. During the course of our study, it was shown that daminozide (also known as Alar), selectively inhibits KDM2/7 demethylases [26]. Daminozide, another 2OG analog and PGR similar to prohexadione, was sprayed on apple trees until 1989, before it was withdrawn due to concerns of its effect on human health. Prohexadione has been classified as a reduced risk pesticide by the Environmental Protection Agency due to its low toxicity and limited persistence in the environment due to photo and microbial degradations [9], [27]. Although it meets the reduced risk criteria for pesticides, our results described in this article indicates that it is essential to set a stringent ‘Maximum Residue Limits’ for prohexadione to promote its safe use for food production. Our results warrant further investigation into the effects of long-term exposure of prohexadione on epigenetic changes associated with neuronal development [25].

## Conflict of interest

The authors declare no conflicts of interest.

## Transparency document

Transparency document

## Figures and Tables

**Figure:**
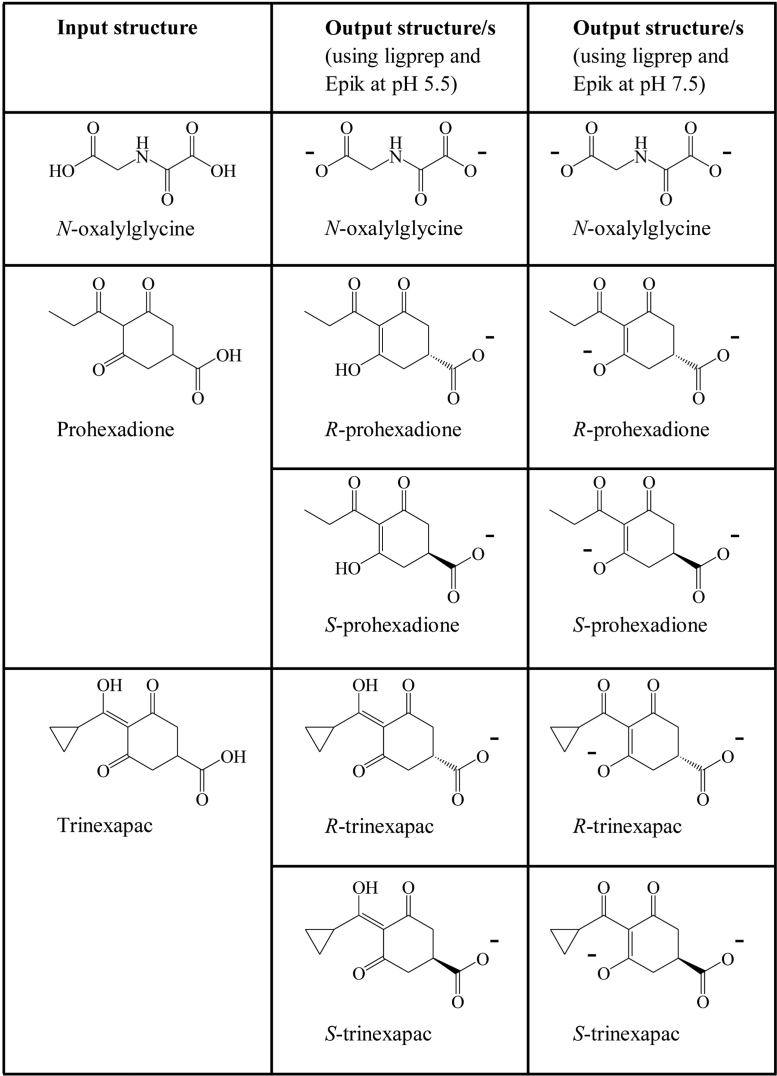


**Figure:**
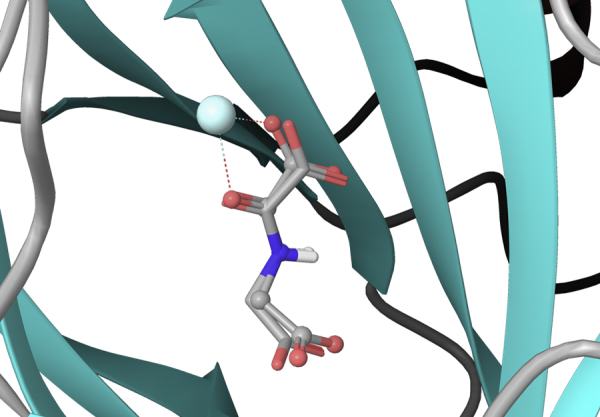


**Figure:**
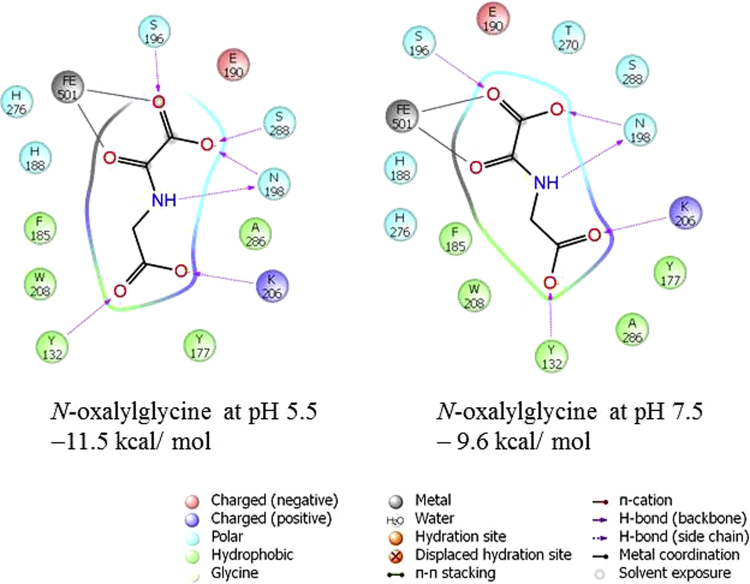


**Figure:**
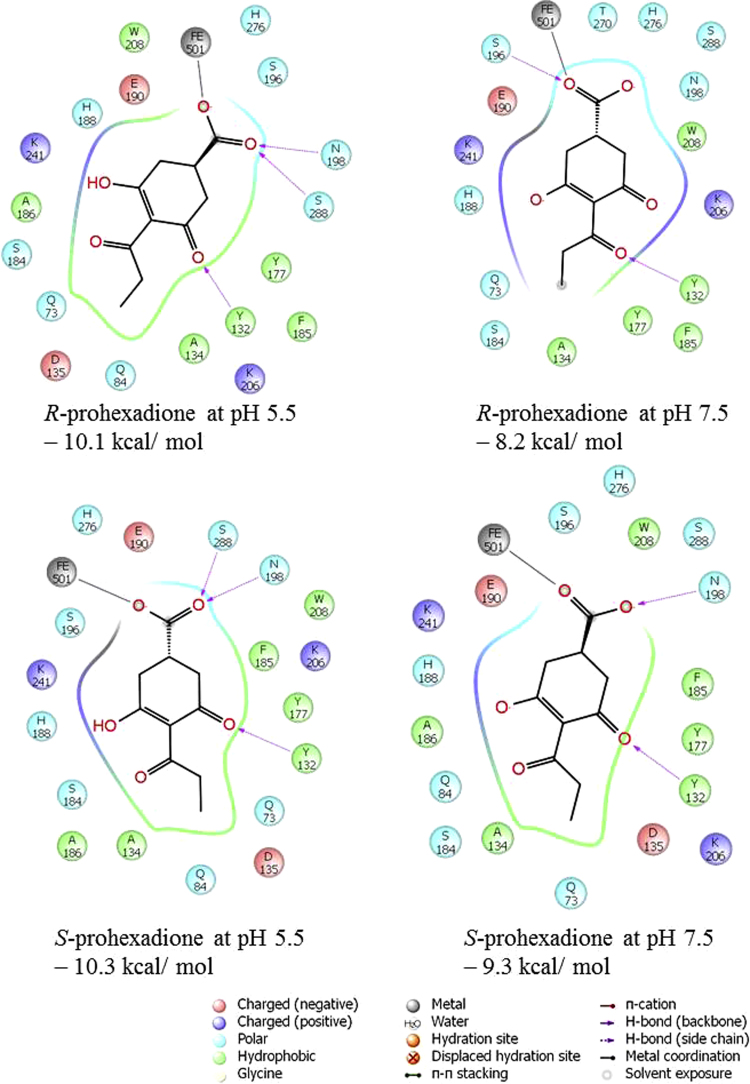


**Figure:**